# Supplementary material for: Causal Relationship between Aging and Anorexia Nervosa: A White-Matter-Microstructure-Mediated Mendelian Randomization Analysis
Source: Biomedicines. 2024 Aug 16;12(8):1874. doi: 10.3390/biomedicines12081874 (PMC11351342; doi:10.3390/biomedicines12081874)
Supplement: Supplementary file 1 [file biomedicines-12-01874-s001.zip › Supplementary Figure/Figure S4/Singe SNP plot of MR analysis for TL to ubm-b-1551.pdf]

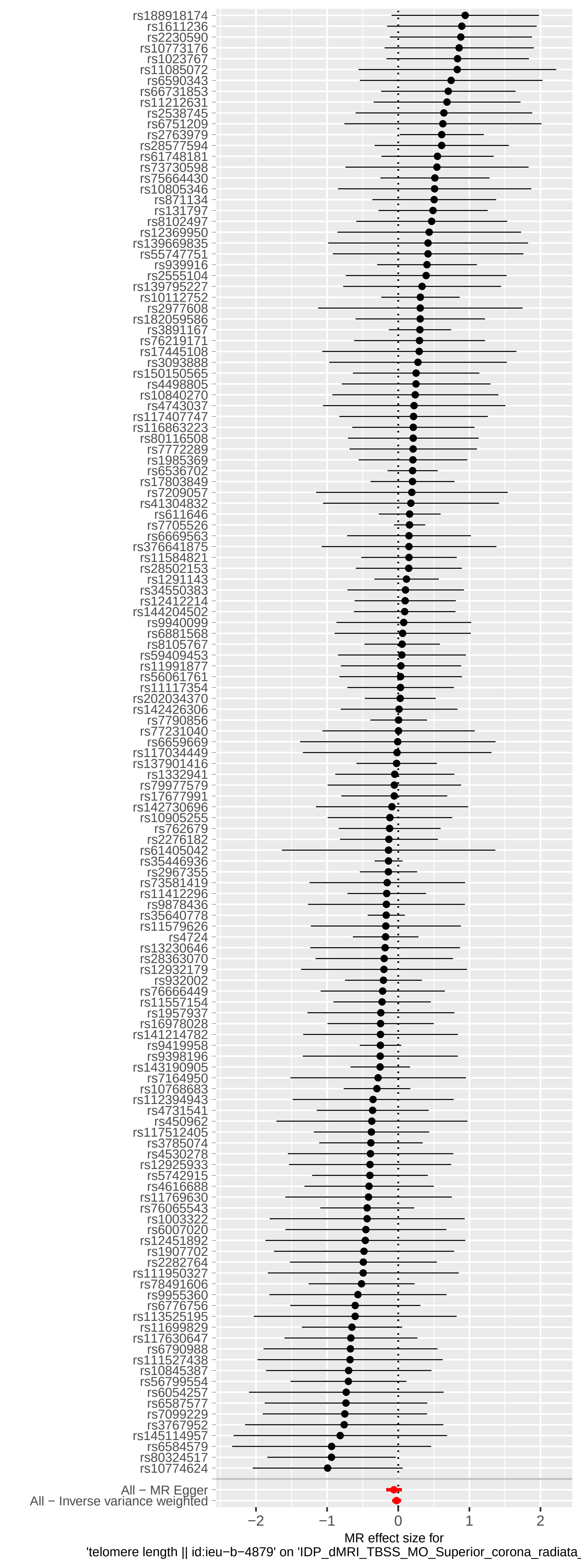

rs188918174
rs1611236
rs2230590
rs10773176
rs1023767
rs11085072
rs6590343
rs66731853
rs11212631
rs2538745
rs6751209
rs2763979
rs28577594
rs61748181
rs73730598
rs75664430
rs10805346
rs871134
rs131797
rs8102497
rs12369950
rs139669835
rs55747751
rs939916
rs2555104
rs139795227
rs10112752
rs2977608
rs182059586
rs3891167
rs76219171
rs17445108
rs3093888
rs150150565
rs4498805
rs10840270
rs4743037
rs117407747
rs116863223
rs80116508
rs7772289
rs1985369
rs6536702
rs17803849
rs7209057
rs41304832
rs611646
rs7705526
rs6669563
rs376641875
rs11584821
rs28502153
rs1291143
rs34550383
rs12412214
rs144204502
rs9940099
rs6881568
rs8105767
rs59409453
rs11991877
rs56061761
rs11117354
rs202034370
rs142426306
rs7790856
rs77231040
rs6659669
rs117034449
rs137901416
rs1332941
rs79977579
rs17677991
rs142730696
rs10905255
rs762679
rs2276182
rs61405042
rs35446936
rs2967355
rs73581419
rs11412296
rs9878436
rs35640778
rs11579626
rs4724
rs13230646
rs28363070
rs12932179
rs932002
rs76666449
rs11557154
rs1957937
rs16978028
rs141214782
rs9419958
rs9398196
rs143190905
rs7164950
rs10768683
rs112394943
rs4731541
rs450962
rs117512405
rs3785074
rs4530278
rs12925933
rs5742915
rs4616688
rs11769630
rs76065543
rs1003322
rs6007020
rs12451892
rs1907702
rs2282764
rs111950327
rs78491606
rs9955360
rs6776756
rs113525195
rs11699829
rs117630647
rs6790988
rs111527438
rs10845387
rs56799554
rs6054257
rs6587577
rs7099229
rs3767952
rs145114957
rs6584579
rs80324517
rs10774624
All − MR Egger
All − Inverse variance weighted
−2
−1
0
1
2
MR effect size for
'telomere length || id:ieu−b−4879' on 'IDP_dMRI_TBSS_MO_Superior_corona_radiata_
